# Oral small-molecule tyrosine kinase 2 and phosphodiesterase 4 inhibitors in plaque psoriasis: a network meta-analysis

**DOI:** 10.3389/fimmu.2023.1180170

**Published:** 2023-06-02

**Authors:** Yuanyuan Xu, Zhixuan Li, Shuwei Wu, Linghong Guo, Xian Jiang

**Affiliations:** ^1^ Department of Dermatology, West China Hospital, Sichuan University, Chengdu, China; ^2^ Laboratory of Dermatology, Clinical Institute of Inflammation and Immunology, Frontiers Science Center for Disease-related Molecular Network, West China Hospital, Sichuan University, Chengdu, China

**Keywords:** psoriasis, tyrosine kinase 2 inhibitor, phosphodiesterase 4 inhibitor, network meta-analysis, treatment strategy

## Abstract

**Background:**

Orally administered small-molecule drugs including tyrosine kinase 2 (TYK2) inhibitors and phosphodiesterase 4 (PDE4) inhibitors are new candidates for systemic therapy in plaque psoriasis. However, no previous articles evaluated the benefit and risk profile of TYK2 and PDE4 inhibitors in psoriasis.

**Objectives:**

The objective of this study was to compare the efficacy and safety of oral small-molecule drugs, including TYK2 and PDE4 inhibitors, in treating moderate-to-severe plaque psoriasis.

**Methods:**

PubMed, Embase, and Cochrane library were searched for eligible randomized clinical trials (RCTs). Response rates for a 75% reduction from baseline in Psoriasis Area and Severity Index (PASI-75) and Physician’s Global Assessment score of 0 or 1 (PGA 0/1) were used for efficacy assessment. Safety was evaluated with the incidence of adverse events (AEs). A Bayesian multiple treatment network meta-analysis (NMA) was performed.

**Results:**

In total, 13 RCTs (five for TYK2 inhibitors and eight for PDE4 inhibitors) involving 5274 patients were included. The study found that deucravacitinib at any dose (except for 3 mg QOD), ropsacitinib (200 and 400 mg QD), and apremilast (20 and 30 mg BID) had higher PASI and PGA response rates than placebo. In addition, deucravacitinib (3 mg BID, 6 mg QD, 6 mg BID, and 12 mg QD), and ropsacitinib (400 mg QD) showed superior efficacy than apremilast (30 mg BID). In terms of safety, deucravacitinib or ropsacitinib at any dose did not lead to a higher incidence of AEs than apremilast (30 mg BID). The ranking analysis of efficacy revealed that deucravacitinib 12 mg QD and deucravacitinib 3 mg BID had the highest chance of being the most effective oral treatment, followed by deucravacitinib 6 mg BID and ropsacitinib 400 mg QD.

**Conclusions:**

Oral TYK2 inhibitors demonstrated satisfactory performance in treating psoriasis, surpassing apremilast at certain doses. More large-scale, long-term studies focusing on novel TYK2 inhibitors are needed.

**Systematic review registration:**

PROSPERO (ID: CRD42022384859), available from: https://www.crd.york.ac.uk/prospero/display_record.php?ID=CRD42022384859, identifier CRD42022384859.

## Introduction

Psoriasis is a chronic, systemic, immune-mediated, inflammatory disorder of skin affecting 2-3% of the global population (1). Characterized by scaly, erythematous patches and/or plaques, plaque-type psoriasis constitutes around 80% of the psoriasis cases (2). Psoriasis significantly impairs the physical and psychological conditions of patients and reduces their health-related quality of life (3). Additionally, psoriasis is associated with the risk of various comorbidities, such as cardiometabolic diseases, gastrointestinal diseases, kidney diseases, malignancies and infections (4).

Various inflammatory cytokines, such as tumor necrosis factor (TNF)-α, interleukin (IL)-12, interleukin (IL)-17, IL-22, and IL-23 and interferon (IFN)-γ, contribute to the immunopathogenesis of psoriasis (5, 6). Treatment options for psoriasis range from conventional therapies, including topical and systemic therapies, to biological therapies. Although biological therapies offer superior efficacy and safety compared to traditional systemic therapies, their use is limited due to loss of response over time, high costs, and problems related to parenteral administration, highlighting the unmet need in psoriasis treatment (7–10). Oral small-molecule inhibitors are a novel group of agents with low molecular weight (<1000 Dalton) that can affect intracellular signaling pathways through the modulation of cytokines (11–13). The promising prospects of oral small molecules in the management of psoriasis is attributed primarily to their simplified synthesis processes, low manufacturing costs, easy administration, and favorable safety profile (12–14).

Currently, oral small molecules available for psoriasis treatment include phosphodiesterase 4 (PDE4) inhibitors and Janus kinase (JAK) inhibitors. PDE4 and JAK are two important classes of molecules involved in the inflammatory process of psoriasis, and are viewed as viable targets for psoriasis therapy (15–17). Apremilast is the only approved oral PDE4 inhibitor by the U.S. Food and Drug Administration (FDA) for psoriasis treatment and is helping to fill an important treatment gap in psoriasis (18, 19). Regarding JAK inhibitors, the limited specificity and therapeutic index of JAK1, 2, and 3 inhibitors have hindered their use in psoriasis treatment. As an alternative, tyrosine kinase 2 (TYK2) inhibitors with enhanced selectivity are recently being investigated as a promising approach (19, 20). Deucravacitinib, an oral, selective TYK2 inhibitor, was granted approval by the FDA in 2022 for treating moderate-to-severe plaque psoriasis in adults (21). Several additional TYK2 inhibitors, including brepocitinib and ropsacitinib, were also under development for treating psoriasis (22–24).

Systemic therapy is an important treatment for psoriasis. Though the molecular mechanisms underlying treatment efficacy are distinguished, oral small-molecule PDE4 and TYK2 inhibitors provide similar advantages in terms of patient convenience, reduced healthcare costs, and improved quality of life (20). No previous studies have ever compared the benefit and risk profile of oral inhibitors of PDE4 and TYK2 in psoriasis treatment. With evidence from randomized controlled trials (RCTs), we aimed to perform a network meta-analysis (NMA) to assess the efficacy and safety of oral PDE4 and TYK2 inhibitors in treating moderate-to-severe plaque psoriasis.

## Methods

This systematic review and network meta-analysis was reported following the guidelines of Preferred Reporting Items for Systematic Reviews and Meta-Analysis (PRISMA) for RCTs. Additionally, the study protocol was registered at PROSPERO (ID: CRD42022384859).

### Data source and search strategy

A systematic search was conducted by two independent investigators (Y.X. and Z.L.) across the PubMed, Embase, and Cochrane library databases until May 10, 2023. The detailed search strategies used by the investigators across different databases are displayed in **Supplementary Table S1**. To expand the scope of relevant data, we performed searches on studies and reviews without any constraints on publication, language, region, or references. After eliminating the repetitive studies, the titles and abstracts of the identified records underwent an initial screening and any discrepancies that arose between the investigators were resolved through discussions with a senior investigator (L.G.).

### Study selection

Studies were considered eligible for inclusion if they met the following criteria: (1) studies that enrolled patients with moderate-to-severe plaque psoriasis; (2) studies that aimed to compare the efficacy or safety profile of any type of oral TYK2 inhibitors or PDE4 inhibitors to placebo; (3) studies that recorded efficacy outcomes including Psoriasis Area and Severity Index (PASI) scores or Physician’s Global Assessment (PGA) scores or safety outcomes including the incidence of adverse events (AEs), with corresponding time points; (4) RCTs. Exclusion criteria were: (1) studies that did not report efficacy outcomes of PASI scores or safety outcomes; (2) studies of observational studies or other types of clinical trials, and studies without complete original data, such as editorials, comments, reviews, protocols, and conference presentations; (3) studies of repetitive publications from the same study group.

### Data extraction

Two investigators (Y.X. and Z.L.) independently conducted the data extraction process and cross-checked the extracted data. The information extracted as baseline characteristics included: study name, study design, type of intervention, the number of patients, sex, age, BMI, disease duration, treatment duration, and outcome measure. In this study, the primary endpoint for efficacy was 75% improvement in the Psoriasis Area and Severity Index (PASI-75) from baseline. The Physician’s Global Assessment achieving “clear” or “almost clear” (PGA 0/1) was also analyzed for efficacy assessment. The incidence of AEs was assessed for safety. Therefore, the number of patients who achieved PASI-75 and PGA 0/1 at the end of the trial and the number of AEs were extracted. Any discrepancies between the investigators during the process of data extraction were resolved through discussions with a senior investigator (L.G.).

### Statistical analysis

All statistical analyses were conducted using the GeMTC package in R software (version 4.0.3). We performed a Bayesian multiple treatment network meta-analysis (NMA) with fixed effects and non-informative priors. An initial burn-in of 20,000 simulations was executed, followed by an additional 50,000 simulations to generate the outputs in each analysis. The convergence of simulations was confirmed with the potential scale reduction factor (PSRF) and Gelman-Rubin-Brooks plots (25). Fixed-effect models were used for the analysis, and additional random-effect models were also applied to evaluate the robustness of the models. Odds ratio (OR) with 95% credibility intervals (CIs) were used to compare the efficacy and safety profile of TYK2 inhibitors, PDE4 inhibitors and placebo in the treatment of plaque psoriasis. The rank of medications with corresponding dosages for each outcome was conducted using the surface under the cumulative ranking curve (SUCRA). For each outcome, SUCRA values range from 0 (being the worst without uncertainty) to 1 (being the best without uncertainty). A two-tailed P value of 0.05 was considered statistically significant.

## Results

### Characteristics of the included studies

**Figure 1:**
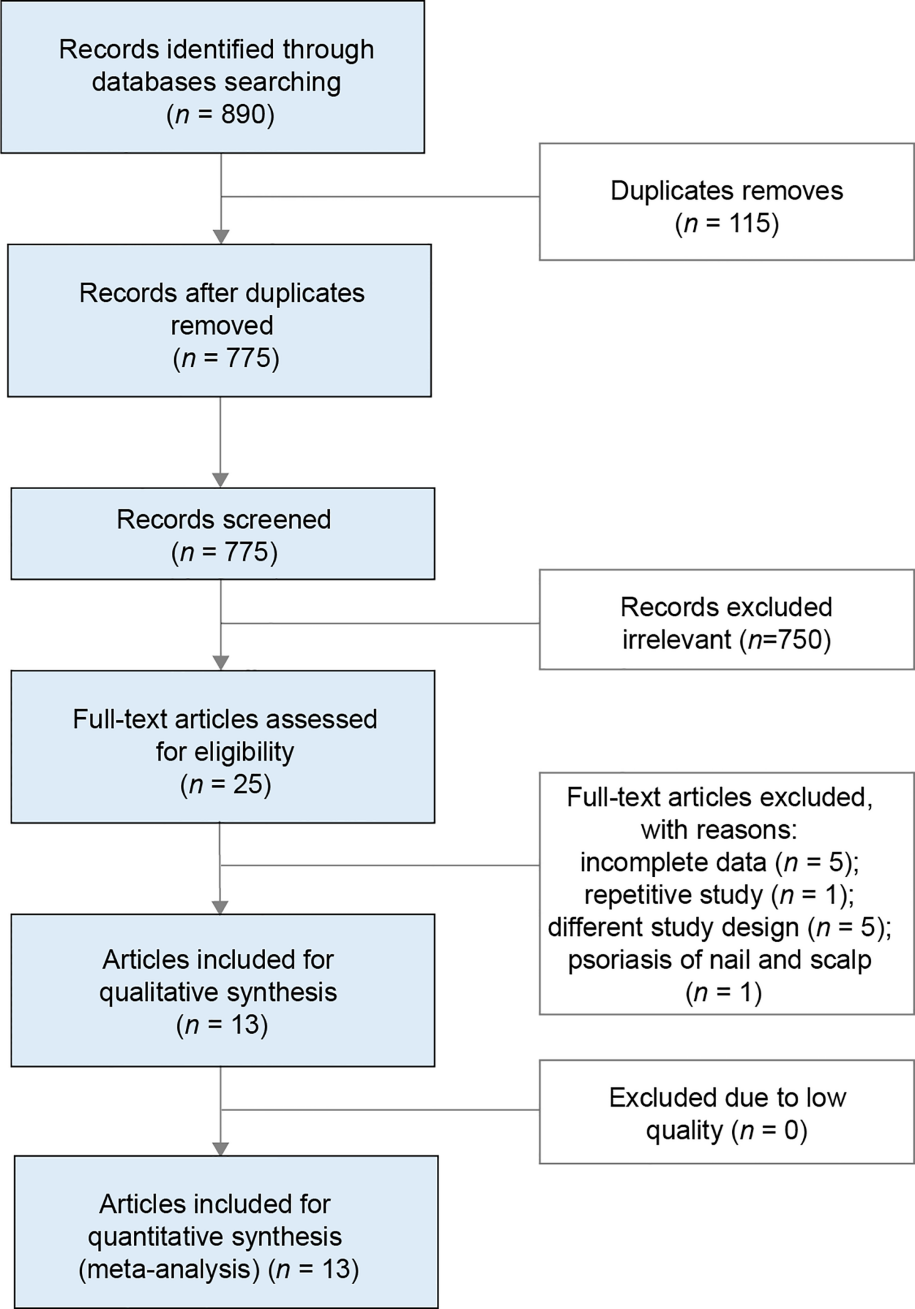
Flow diagram of the search and screening of the literature.

The systematic search across multiple databases yielded 890 records, of which 775 articles remained after removing duplicates. Screening of titles and abstracts led to the exclusion of 750 articles, leaving 25 records for full-text review. Of these, 13 were considered eligible for the final analysis, comprising eight articles on oral PDE4 inhibitors (26–33), and five articles on oral TYK2 inhibitors (23, 24, 34–36). The flow of literature selection is presented in **Figure 1**.

**Table 1** presents a summary of the characteristics of the studies and patients included in the NMA. The sample size of the 13 included RCTs ranged from 40 to 1020, with 12 (92%) containing more than 100 patients. The included studies were composed of one Phase IV study, six Phase III studies, five Phase II studies, and one Phase I study. Particularly, large Phase III study data were available for deucravacitinib and apremilast. The five articles on oral TYK2 inhibitors were mostly published between 2021 and 2022, with three focusing on deucravacitinib and two on ropsacitinib. The eight studies concerning the oral PDE4 inhibitor were published between 2012 and 2022, and all of them investigated the efficacy and safety profile of apremilast. All of the 13 eligible studies enrolled adult patients with moderate-to-severe plaque psoriasis, and 10 of them had a treatment duration of 16 weeks (77%).

**Table 1:** Characteristics of included studies in this meta-analysis.

Study name	Treatment and dose	No. of patients	Female, no. (%)	Age, year (mean, SD)	BMI (mean, SD)	Disease duration (mean, SD)	End point	Efficacy outcome measure	Study design
Armstrong et al. 2022 (NCT03624127)	Deucravacitinib 6 mg QD	332	102 (30.7)	45.9 (13.7)	29.8 (7.0)	17.1 (12.4)	16w	PASI-75/90/100 PGA 0/1 scPGA 0/1 DLQI	Phase III
	Apremilast 30 mg BID	168	58 (34.5)	44.7 (12.1)	29.6 (6.7)	17.7 (11.8)			
	Placebo	166	53 (31.9)	47.9 (14.0)	30.2 (7.4)	17.3 (12.8)			
Gold et al., 2022 (NCT03721172)	Apremilast 30 mg BID	297	123 (41.4)	49.2 (14.7)	31.2 (7.2)	16.9 (14.3)	16w	PGA 0/1 BSA-75 WBI-NRS response scPGA 0/1 DLQI	Phase III
	Placebo	298	147 (49.3)	48.3 (14.5)	30.9 (7.0)	16.9 (13.9)			
Ohtsuki et al., 2017 (NCT01988103)	Apremilast 20 mg BID	85	16 (18.8)	52.2 (12.5)	25.8 (4.2)	12.6 (10.6)	16w	PASI-50/75/90 PGA 0/1 DLQI pruritus VAS	Phase III
	Apremilast 30 mg BID	85	14 (16.5)	51.7 (12.7)	24.9 (3.7)	13.9 (9.2)			
	Placebo	84	22 (26.2)	48.3 (12.0)	24.7 (4.7)	12.4 (9.4)			
Papp et al., 2012 (NCT00773734)	Apremilast 10 mg BID	89	26 (29.2)	44.4 (13.9)	32.5 (7.4)	18.0 (12.4)	16w	PASI-50/75/90 PGA 0/1 BSA pruritus VAS DLQI	Phase IIb
	Apremilast 20 mg BID	87	32 (36.8)	44.6 (12.6)	30.4 (6.1)	19.2 (12.1)			
	Apremilast 30 mg BID	88	38 (43.2)	44.1 (14.7)	31.1 (7.7)	19.2 (12.0)			
	Placebo	88	35 (39.8)	44.1 (13.7)	30.8 (6.7)	19.6 (11.6)			
Papp et al., 2013 (NCT00606450)	Apremilast 20 mg QD	87	26 (29.9)	46.2 (11.8)	NA	19.1 (12.0)	12w	PASI-50/75/90 PGA BSA	Phase II
	Apremilast 20 mg BID	85	36 (42.4)	48.4 (12.3)	NA	20.7 (13.3)			
	Placebo	87	34 (39.1)	43.7 (12.4)	NA	17.6 (11.8)			
Papp 2015 (NCT01194219)	Apremilast 30 mg BID	562	183 (32.6)	45.8 (13.1)	31.2 (6.7)	19.8 (13.0)	16w	PASI-50/75/90 PGA 0/1 DLQI pruritus VAS	Phase III
	Placebo	282	88 (31.2)	46.5 (12.7)	31.3 (7.4)	18.7 (12.4)			
Papp et al., 2018 (NCT02931838)	Deucravacitinib 3 mg QOD	44	8 (18.1)	41 (12)	29 (6)	18 (1–52)	12w	PASI-50/75/90; PGA DLQI	Phase II
	Deucravacitinib 3 mg QD	44	14 (31.8)	45 (14)	29 (5)	13 (2–60)			
	Deucravacitinib 3 mg BID	45	19 (42.2)	46 (15)	28 (5)	13 (1–61)			
	Deucravacitinib 6 mg BID	45	10 (22.2)	43 (13)	27 (5)	15 (1–55)			
	Deucravacitinib 12 mg QD	44	14 (31.8)	47 (12)	29 (5)	20 (1–47)			
	Placebo	45	8 (17.8)	46 (12)	30 (6)	18 (2–48)			
Paul et al., 2015 (NCT01232283)	Apremilast 30 mg BID	274	98 (35.8)	45.3 (13.1)	30.9 (6.7)	17.9 (11.4)	16w	PASI-50/75/90 PGA 0/1 DLQI pruritus VAS	Phase III
	Placebo	137	37 (27.0)	45.7 (13.4)	30.7 (7.1)	18.7 (12.1)			
Reich et al., 2017 (NCT01690299)	Apremilast 30 mg BID	83	34 (41.0)	46.0 (13.6)	29.2 (5.8)	19.7 (12.7)	16w	PASI-50/75/90 PGA 0/1 DLQI pruritus VAS	Phase IIIb
	Placebo	84	25 (29.8)	43.4 (14.9)	29.5 (6.6)	16.6 (12.1)			
Strober et al., 2017 (NCT02425826)	Apremilast 30 mg BID	148	74 (50.0)	48.6 (15.4)	30.5 (7.4)	17.5 (13.9)	16w	PGA x BSA PGA 0/1 PtGA 0/1 PASI-75 DLQI	Phase IV
	Placebo	73	32 (43.8)	51.1 (13.7)	30.8 (6.4)	13.9 (12.6)			
Strober et al., 2022 (NCT03611751)	Deucravacitinib 6 mg QD	511	175 (34.2)	46.9 (13.4)	31.0 (6.8)	19.6 (12.9)	16w	PASI-75/90/100 PGA 0/1 ss-PGA 0/1 DLQI	Phase III
	Apremilast 30 mg BID	254	97 (38.2)	46.4 (13.3)	31.6 (7.2)	18.9 (12.4)			
	Placebo	255	74 (29.0)	47.3 (13.6)	30.4 (6.3)	19.9 (12.9)			
Tehlirian et al., 2021 (NCT03210961)	Ropsacitinib 100 mg QD	11	0	39.0 (13.39)	NA	9.6 (10.9)	4w	PASI-75/90/100 PGA 0/1 BSA TPSS	Phase I
	Ropsacitinib 400 mg QD	15	0	40.9 (11.62)	NA	17.8 (16.8)			
	Placebo	14	0	38.3 (13.25)	NA	10.5 (19.2)			
Tehlirian et al., 2022 (NCT03895372)	Ropsacitinib 50 mg QD	22	7 (31.8)	43.1 (14.5)	NA	NA	16w	PASI-75/90/100 PGA 0/1 NRS	Phase IIb
	Ropsacitinib 100 mg QD	23	7 (30.4)	41.8 (12.4)	NA	NA			
	Ropsacitinib 200 mg QD	45	19 (42.2)	45.0 (13.0)	NA	NA			
	Ropsacitinib 400 mg QD	43	8 (18.6)	45.2 (12.2)	NA	NA			
	Placebo	45	15 (33.3)	46.5 (12.1)	NA	NA			

BSA, body surface area; DLQI, dermatology life quality index; NRS, numerical rating scale; PASI, psoriasis area and severity index; PGA, physician’s global assessment; PtGA, patient global assessment; scPGA, static physician’s global assessment; TPSS, total psoriasis severity score; VAS, visual analog scale; NA, not available.

The Cochrane Collaboration’s tool for assessing the risk of bias in randomized trials was used to evaluate the quality of the studies. As a result, all of the trials included were deemed to have an acceptable risk of bias and were qualified for further analysis. More details on the quality assessment can be found in **Figure S1**.

### Network meta-analysis of PASI response

**Figure 2:**
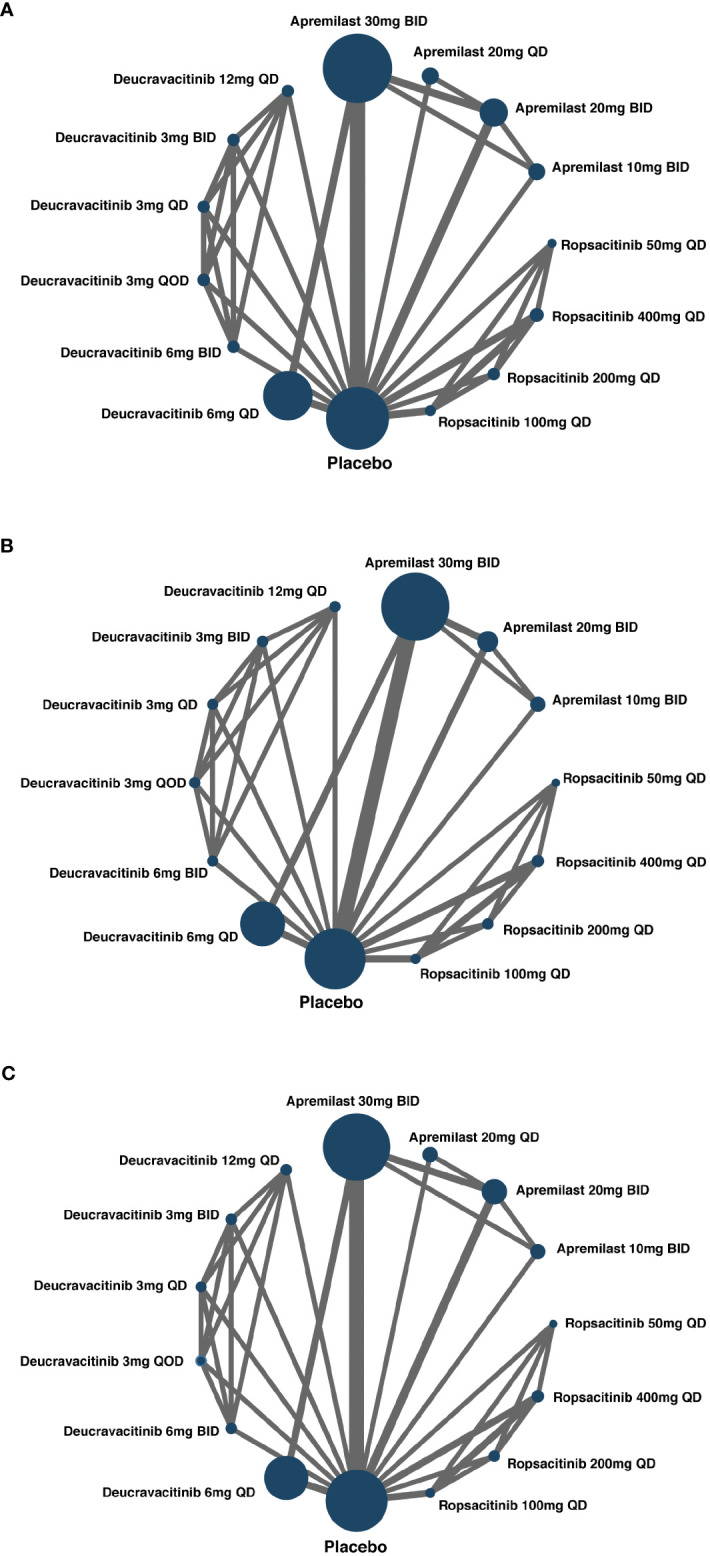
Network of included studies with the available direct comparisons. The size of the nodes is proportional to the number of patients to receive the treatment. The width of the lines is proportional to the number of trials comparing the connected treatments. **(A)** For PASI-75 response direct comparisons; **(B)** for PGA 0/1 response direct comparisons; **(C)** for AEs direct comparisons.

Except for one study (26), the other 12 eligible studies provided data on the number of patients who attained a PASI-75 response after the duration of treatment. A network plot was generated to visualize the networks of comparisons between different treatments regarding PASI-75 response (**Figure 2A**).

**Figure 3:**
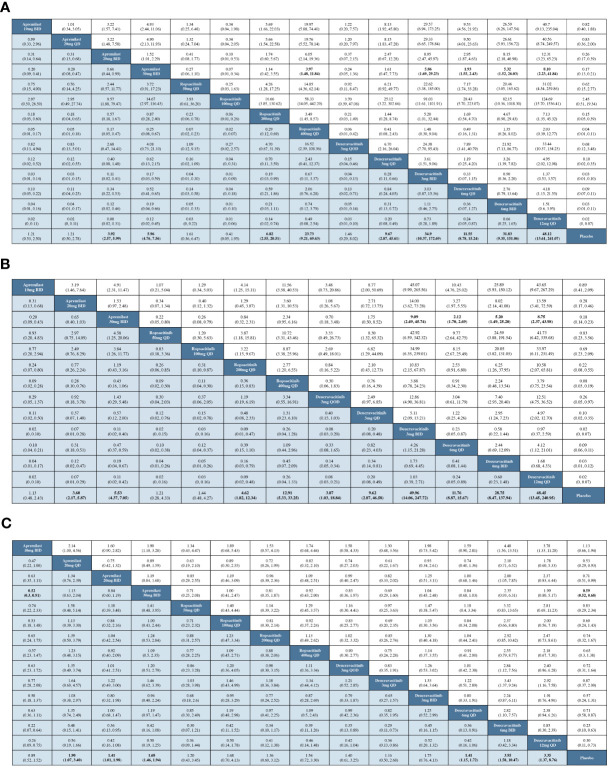
Pairwise comparisons by OR and corresponding 95% CI. **(A)** For the PASI-75 response; **(B)** for the PGA 0/1 response; **(C)** for the incidence of AEs.

The result of the NMA revealed that, in comparison to placebo, the low-dose groups of oral TYK2 and PDE4 inhibitors did not exhibit superior efficacy. However, when administered at sufficient dosages, the oral TYK2 and PDE4 inhibitors displayed significantly greater PASI-75 response rates than placebo. Notably, apremilast 20 or 30 mg BID, ropsacitinib 200 or 400 mg QD, and deucravacitinib at all dosages except for 3 mg QOD exhibited significantly greater efficacy than placebo. Moreover, when compared to apremilast 30 mg BID, the NMA demonstrated that oral TYK2 inhibitors, specifically deucravacitinib 3 mg BID, deucravacitinib 6 mg QD, deucravacitinib 6 mg BID, deucravacitinib 12 mg QD, and ropsacitinib 400 mg QD, displayed significantly higher efficacy, as shown in the forest plot (**Figure S2**). **Figure 3A** presents the pairwise comparisons of PASI-75 response rates between TYK2, PDE4 inhibitors, and placebo. This finding highlights the potential of novel TYK2 inhibitors as an effective oral medication for the treatment of psoriasis.

**Figure 4:**
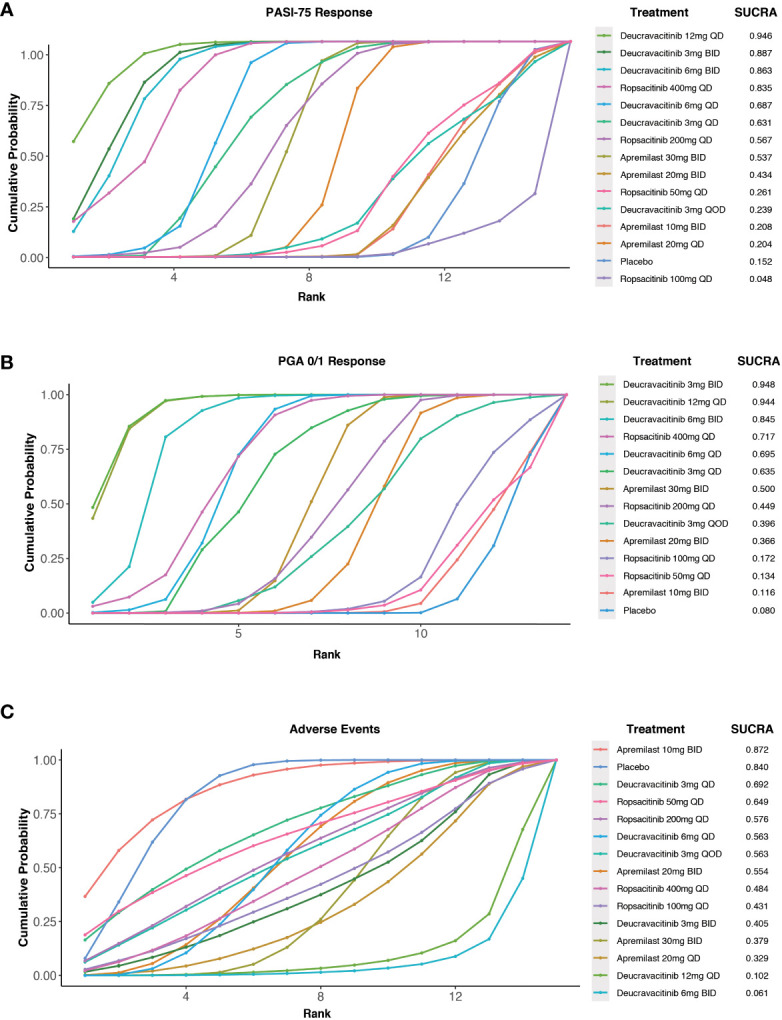
Surface under the cumulative ranking curve (SUCRA) probabilities of different medications for efficacy and safety outcomes. **(A)** For the PASI-75 response; **(B)** for the PGA 0/1 response; **(C)** for the incidence of AEs.

According to the efficacy ranking analysis, among all the TYK2 and PDE4 inhibitors included in our study, deucravacitinib 12 mg QD demonstrated the highest cumulative probability of achieving a PASI-75 response (SUCRA value = 0.946). This was followed by deucravacitinib 3 mg BID (SUCRA value = 0.887), deucravacitinib 6 mg BID (SUCRA value = 0.863), and ropsacitinib 400 mg QD (SUCRA value = 0.835) (**Figure 4A**).

### Network meta-analysis of PGA response

With data from 12 studies, the NMA was conducted to evaluate the efficacy of different oral treatments on achieving a PGA 0/1 response. The comparison networks for PGA 0/1 response is illustrated in a network plot (**Figure 2B**). This NMA result revealed that deucravacitinib at all doses, apremilast 20 or 30 mg BID, and ropsacitinib 200 or 400 mg QD were significantly more effective than placebo in treating psoriasis. Additionally, deucravacitinib 3 mg BID, deucravacitinib 6 mg QD, deucravacitinib 6 mg BID, and deucravacitinib 12 mg QD displayed greater efficacy than apremilast 30 mg BID, as shown in the forest plot (**Figure S3**). **Figure 3B** presents the pairwise comparisons of PGA 0/1 response rates between the TYK2, PDE4 inhibitors, and placebo. The consistency of results between NMA based on PASI-75 or PGA 0/1 response was mostly observed, with some subtle statistical differences.

A cumulative ranking analysis by PGA response was performed, and the results indicated that deucravacitinib 12 mg QD (SUCRA value = 0.944) and deucravacitinib 3 mg BID (SUCRA value = 0.948) had the highest chance of being the most effective oral treatment options, which were followed by deucravacitinib 6 mg BID (SUCRA value = 0.845) and ropsacitinib 400 mg QD (SUCRA value = 0.717) (**Figure 4B**).

### Network meta-analysis of AEs

All RCTs included in this study provided information on AE incidence during the treatment. Networks of comparisons for AEs between TYK2, PDE4 inhibitors, and placebo can be found in **Figure 2C**. Based on the NMA, it was observed that apremilast 20 mg QD, apremilast 20 mg BID, apremilast 30 mg BID, deucravacitinib 6 mg QD, deucravacitinib 6 mg BID, and deucravacitinib 12 mg QD had a higher incidence of AEs when compared to placebo. The NMA additionally suggested that ropsacitinib or deucravacitinib with all dosages did not show higher risk for AEs compared to apremilast 30 mg BID, as indicated in the forest plot (**Figure S4**). The pairwise comparisons of the incidence of AEs between TYK2, PDE4 inhibitors, and placebo are displayed in **Figure 3C**.

The analysis of safety ranking revealed that patients treated with apremilast 10 mg BID had the least chance of AE occurrence among the oral medications (SUCRA value = 0.872), followed by deucravacitinib 3 mg QD (SUCRA value = 0.692) and ropsacitinib 50 mg QD (SUCRA value = 0.649) (**Figure 4C**).

### Sensitivity analyses

In order to assess the robustness of the NMA results, we conducted sensitivity analyses. The results were consistent with both fixed-effect and random-effect models, as presented in **Supplementary Table S2**. This indicates that our conclusions are robust and reliable across the range of studies analyzed.

## Discussion

### Principle findings

The NMA findings revealed that deucravacitinib, except for the 3 mg QOD dose, ropsacitinib 200 and 400 mg QD, and apremilast 20 and 30 mg BID, resulted in significantly higher PASI-75 and PGA 0/1 response rates compared to placebo. In addition, deucravacitinib at 3 mg BID, 6 mg QD, 6 mg BID, 12 mg QD, and ropsacitinib 400 mg QD showed better efficacy response than apremilast 30 mg BID, which is the recommended maintenance dosage for most psoriasis patients who are treated with apremilast (37). In terms of AEs, deucravacitinib and ropsacitinib at any dose did not demonstrate a higher incidence of AEs than apremilast 30 mg BID. The ranking analysis based on PASI and PGA revealed that deucravacitinib 12 mg QD and deucravacitinib 3 mg BID had the highest chance of being the most effective oral treatment, followed by deucravacitinib 6 mg BID and ropsacitinib 400 mg QD. Overall, these findings suggested that, like apremilast, a PDE4 inhibitor that has been widely used as a systemic drug for psoriasis since its approval in 2014, TYK2 inhibitors also have the potential to serve as a type of novel medications for the systemic treatment of psoriasis, holding substantial promise in filling the existing treatment gaps.

### Comparison with previous studies

To the best of our knowledge, this systematic review and network meta-analysis represents the first study to assess and compare the efficacy and safety profile of oral PDE4 and TYK2 inhibitors in the treatment of plaque psoriasis. A previous meta-analysis compared the efficacy and safety of JAK inhibitors in treating plaque psoriasis, and concluded that tofacitinib had a better performance in efficacy and safety than peficitinib, solcitinib, baricitinib, abrocitinib and deucravacitinib (38). However, the FDA declined to approve tofacitinib 10 mg BID for plaque psoriasis treatment owing to enhanced risk of AEs, leading Pfizer to discontinue its development for this indication (20). Furthermore, the aforementioned network meta-analysis included only one Phase II clinical trials concerning deucravacitinib (35), while our study additionally incorporated two large-scale Phase III clinical trials from 2022 to provide a more comprehensive comparison of its efficacy against apremilast and placebo (34, 36).

Our result was in consistency with the two large and recent Phase III clinical trials that compared the efficacy and safety of deucravacitinib 6 mg QD with apremilast 30 mg BID and placebo, in which the researchers demonstrated that deucravacitinib 6 mg QD was associated with higher PASI-75 (58.4% vs. 35.1%) and PGA 0/1 (53.6% vs. 32.1%) response rate at week 16 (34, 36). Our study further indicated that deucravacitinib, in various combinations of dosage and administration frequency (3 mg BID, 6 mg QD, 6 mg BID, and 12 mg QD), continued to exhibit superior efficacy assessed by PASI-75 and PGA 0/1 response rate when compared to apremilast 30 mg BID. Another Phase IIb clinical trial demonstrated that ropsacitinib (200 mg QD and 400 mg QD) had better efficacy assessed by PASI-75 response compared to placebo (46.7% vs. 13.3%, 73.2% vs. 13.3%, respectively), however, it was not compared with the efficacy of apremilast at 30 mg BID (23). Similarly, our findings revealed that ropsacitinib (200 mg QD and 400 mg QD) was superior in efficacy of PASI response versus placebo (OR: 6.82, 95%Cl: 2.55–20.51; OR: 23.73, 95%Cl: 9.21–69.63), and further indicated that ropsacitinib 400 mg QD had higher PASI response rate than apremilast 30 mg BID (OR: 3.97, 95%Cl: 1.48–11.84).

### Underlying mechanism

Oral small-molecule PDE4 and TYK2 inhibitors appear effective and safe in treating psoriasis, but they work through different mechanisms of action. PDE4 is a major class of enzyme capable of hydrolyzing cyclic adenosine monophosphate (cAMP), a key “secondary messenger” that can modulate the cellular immune response by regulating the network of pro-inflammatory and anti-inflammatory cytokines (39). PDE4 inhibitors can elevate intracellular cAMP, which downregulates inflammation by blocking pro-inflammatory cytokines, including TNF-α, IL-23, and IFN-γ, and increasing anti-inflammatory cytokines, such as IL-10 (5, 11, 40, 41).

On the other hand, JAKs, comprising JAK1, JAK2, JAK3, and TYK2, function as important signal transducers in intracellular signaling pathways stimulated by cytokines (42). The binding of cytokines to their cognate receptors on the cell membrane induces the activation and phosphorylation of JAKs and the phosphorylation of intracellular receptor segments. This cascade results in the phosphorylation and dimerization of signal transducer and activator of transcription (STAT) proteins, which subsequently relocate to the nucleus and modulate gene transcription (43, 44). The above-mentioned JAK-STAT signaling plays a crucial role in various physiological processes: JAK1, 2, and 3 are involved in transmitting signals that regulate a broad range of systemic responses, such as hematopoiesis, myelopoiesis, lipid metabolism, and bone homeostasis. In contrast, TYK2 nearly exclusively participates in immune cytokine signaling pathways, particularly IFN-α, IL-12, and IL-23 (45, 46). Consequently, the clinical investigation of JAK1, 2, and 3 inhibitors for psoriasis therapy has been largely discontinued because of a low therapeutic index and increased safety concerns, including increased risk of infection, herpes zoster, neutropenia, and abnormal laboratory results; while TYK2 inhibitors, as a class of more selective JAK inhibitors, may become promising candidate for psoriasis treatment (20, 45, 47). Deucravacitinib, a type of TYK2 inhibitor, can potentially exhibit superior efficacy and safety compared to other JAK inhibitors in psoriasis treatment, possibly owing to its more specific and targeted mechanism of action in immune response.

### Limitations

This study has several limitations. First, a relatively small number of patients using ropsacitinib were available for our analysis, leading to wider confidence intervals for the corresponding results. In addition, a clinical trial on brepocitinib was excluded from the analysis due to differences in study design and a small number of participants, resulting in an inability to compare the efficacy and safety profile of brepocitinib with other TYK2 and PDE4 inhibitors (22). Therefore, more large-scale clinical trials investigating novel TYK2 inhibitors like ropsacitinib and brepocitinib are needed. Secondly, most of the RCTs included had a treatment duration of 16 weeks, precluding assessment of the long-term performance of these oral small-molecule drugs. More long-term studies are necessary to learn the efficacy and safety of PDE4 and TYK2 inhibitors for psoriasis when being used for a long period of time. Finally, higher treatment goals such as PASI-90 and PASI-100 response rates and treatment effects in specific areas such as nails, scalp, and palms were not analyzed. Hence, studies focusing on difficult-to-treat areas with more comprehensive data on efficacy outcomes are in demand.

## Conclusions

In conclusion, this NMA has validated the potential of orally administered small-molecule drugs, including PDE4 and TYK2 inhibitors, in the treatment of moderate-to-severe plaque psoriasis. Oral TYK2 inhibitors have displayed promising efficacy and safety profiles, surpassing oral PDE4 inhibitors at certain doses. Specifically, deucravacitinib at various doses (3 mg BID, 6 mg QD, 6 mg BID, 12 mg QD) and ropsacitinib (400 mg QD) were more effective than apremilast (30 mg BID) in treating plaque psoriasis, with no increased risk of AEs reported at any dosage. Therefore, TYK2 inhibitors have demonstrated satisfactory performance in treating plaque psoriasis, warranting further development and more long-term, large-scale clinical trials for novel TYK2 inhibitors.

## Data availability statement

The original contributions presented in the study are included in the article and **Supplementary Material**. Further inquiries can be directed to the corresponding authors.

## Author contributions

All authors contributed to the article and approved the submitted version. YX and ZL searched the literature and extracted the data. YX and SW performed the statistical analyses. YX wrote the article. LG and XJ reviewed and revised this article.

## Supplementary material

Supplementary Figure 1 Summary of risk of bias: review authors’ judgements about each risk of bias item for each included study.

Supplementary Figure 2 Forest plot of the PASI-75 response.

Supplementary Figure 3 Forest plot of the PGA 0/1 response.

Supplementary Figure 4 Forest plot of the incidence of AEs.

## References

[1] Greb JE Goldminz AM Elder JT Lebwohl MG Gladman DD Wu JJ . Psoriasis. Nat Rev Dis Primers (2016) 2:16082. doi: 10.1038/nrdp.2016.82 27883001

[2] Armstrong AW Read C . Pathophysiology, clinical presentation, and treatment of psoriasis: a review. Jama (2020) 323(19):1945–60. doi: 10.1001/jama.2020.4006 32427307

[3] Mattei PL Corey KC Kimball AB . Cumulative life course impairment: evidence for psoriasis. Curr Probl Dermatol (2013) 44:82–90. doi: 10.1159/000350008 23796812

[4] Takeshita J Grewal S Langan SM Mehta NN Ogdie A Van Voorhees AS . Psoriasis and comorbid diseases: epidemiology. J Am Acad Dermatol (2017) 76(3):377–90. doi: 10.1016/j.jaad.2016.07.064 PMC5731650 28212759

[5] Gooderham M Papp K . Selective phosphodiesterase inhibitors for psoriasis: focus on apremilast. BioDrugs (2015) 29(5):327–39. doi: 10.1007/s40259-015-0144-3 PMC4626529 26481941

[6] Wittmann M Helliwell PS . Phosphodiesterase 4 inhibition in the treatment of psoriasis, psoriatic arthritis and other chronic inflammatory diseases. Dermatol Ther (Heidelb) (2013) 3(1):1–15. doi: 10.1007/s13555-013-0023-0 23888251 PMC3680635

[7] Au SC Madani A Alhaddad M Alkofide M and Gottlieb AB . Comparison of the efficacy of biologics versus conventional systemic therapies in the treatment of psoriasis at a comprehensive psoriasis care center. J Drugs Dermatol (2013) 12(8):861–6. 23986158

[8] Levin AA Gottlieb AB Au SC . A comparison of psoriasis drug failure rates and reasons for discontinuation in biologics vs conventional systemic therapies. J Drugs Dermatol (2014) 13(7):848–53. 25007369

[9] Garber C Plotnikova N Au SC Sorensen EP Gottlieb A . Biologic and conventional systemic therapies show similar safety and efficacy in elderly and adult patients with moderate to severe psoriasis. J Drugs Dermatol (2015) 14(8):846–52. 26267729

[10] Kragballe K van de Kerkhof PC Gordon KB . Unmet needs in the treatment of psoriasis. Eur J Dermatol (2014) 24(5):523–32. doi: 10.1684/ejd.2014.2403 25115238

[11] Torres T Filipe P . Small molecules in the treatment of psoriasis. Drug Dev Res (2015) 76(5):215–27. doi: 10.1002/ddr.21263 26255795

[12] Afra TP Razmi TM Dogra S . Apremilast in psoriasis and beyond: big hopes on a small molecule. Indian Dermatol Online J (2019) 10(1):1–12. doi: 10.4103/idoj.IDOJ_437_18 30775293 PMC6362739

[13] Sernicola A Russo I Alaibac M . Small-Molecule-Based immunotherapy for immunologically mediated skin conditions. Immunotherapy (2020) 12(6):417–29. doi: 10.2217/imt-2019-0190 32308089

[14] Chen WJ Peng C Lu JJ Ding YF Li XZ . Advances in small molecule inhibitors for treatment of psoriasis. Chin Med J (Engl) (2021) 134(11):1364–6. doi: 10.1097/cm9.0000000000001351 PMC8183699 34075903

[15] Milakovic M Gooderham MJ . Phosphodiesterase-4 inhibition in psoriasis. Psoriasis (Auckl) (2021) 11:21–9. doi: 10.2147/ptt.S303634 PMC7982714 33763335

[16] Martin G . Novel therapies in plaque psoriasis: a review of tyrosine kinase 2 inhibitors. Dermatol Ther (Heidelb) (2023) 13(2):417–35. doi: 10.1007/s13555-022-00878-9 PMC9884727 36592300

[17] Krueger JG McInnes IB Blauvelt A . Tyrosine kinase 2 and janus Kinase−Signal transducer and activator of transcription signaling and inhibition in plaque psoriasis. J Am Acad Dermatol (2022) 86(1):148–57. doi: 10.1016/j.jaad.2021.06.869 34224773

[18] Poole RM Ballantyne AD . Apremilast: first global approval. Drugs (2014) 74(7):825–37. doi: 10.1007/s40265-014-0218-4 24797159

[19] Vangipuram R Alikhan A . Apremilast for the management of moderate to severe plaque psoriasis. Expert Rev Clin Pharmacol (2017) 10(4):349–60. doi: 10.1080/17512433.2017.1293519 28276777

[20] Nogueira M Puig L Torres T . Jak inhibitors for treatment of psoriasis: focus on selective Tyk2 inhibitors. Drugs (2020) 80(4):341–52. doi: 10.1007/s40265-020-01261-8 32020553

[21] Hoy SM . Deucravacitinib: first approval. Drugs (2022) 82(17):1671–9. doi: 10.1007/s40265-022-01796-y PMC9676857 36401743

[22] Forman SB Pariser DM Poulin Y Vincent MS Gilbert SA Kieras EM . Tyk2/Jak1 inhibitor pf-06700841 in patients with plaque psoriasis: phase iia, randomized, double-blind, placebo-controlled trial. J Invest Dermatol (2020) 140(12):2359–70.e5. doi: 10.1016/j.jid.2020.03.962 32311398

[23] Tehlirian C Singh RSP Pradhan V Roberts ES Tarabar S Peeva E . Oral tyrosine kinase 2 inhibitor pf-06826647 demonstrates efficacy and an acceptable safety profile in participants with moderate-to-Severe plaque psoriasis in a phase 2b, randomized, double-blind, placebo-controlled study. J Am Acad Dermatol (2022) 87(2):333–42. doi: 10.1016/j.jaad.2022.03.059 35398218

[24] Tehlirian C Peeva E Kieras E Scaramozza M Roberts ES Singh RSP . Safety, tolerability, efficacy, pharmacokinetics, and pharmacodynamics of the oral Tyk2 inhibitor pf-06826647 in participants with plaque psoriasis: a phase 1, randomised, double-blind, placebo-controlled, parallel-group study. Lancet Rheumatol (2021) 3(3):e204–e13. doi: 10.1016/S2665-9913(20)30397-0 38279383

[25] Brooks SP Gelman AE . General methods for monitoring convergence of iterative simulations. J Comput Graphical Stat (1997) 7(4):434–455. doi: 10.2307/1390675

[26] Stein Gold L Papp K Pariser D Green L Bhatia N Sofen H . Efficacy and safety of apremilast in patients with mild-to-Moderate plaque psoriasis: results of a phase 3, multicenter, randomized, double-blind, placebo-controlled trial. J Am Acad Dermatol (2022) 86(1):77–85. doi: 10.1016/j.jaad.2021.07.040 34343599

[27] Papp K Cather JC Rosoph L Sofen H Langley RG Matheson RT . Efficacy of apremilast in the treatment of moderate to severe psoriasis: a randomised controlled trial. Lancet (2012) 380(9843):738–46. doi: 10.1016/s0140-6736(12)60642-4 22748702

[28] Papp KA Kaufmann R Thaçi D Hu C Sutherland D Rohane P . Efficacy and safety of apremilast in subjects with moderate to severe plaque psoriasis: results from a phase ii, multicenter, randomized, double-blind, placebo-controlled, parallel-group, dose-comparison study. J Eur Acad Dermatol Venereol (2013) 27(3):e376–83. doi: 10.1111/j.1468-3083.2012.04716.x 23030767

[29] Papp K Reich K Leonardi CL Kircik L Chimenti S Langley RG . Apremilast, an oral phosphodiesterase 4 (Pde4) inhibitor, in patients with moderate to severe plaque psoriasis: results of a phase iii, randomized, controlled trial (Efficacy and safety trial evaluating the effects of apremilast in psoriasis [Esteem] 1). J Am Acad Dermatol (2015) 73(1):37–49. doi: 10.1016/j.jaad.2015.03.049 26089047

[30] Paul C Cather J Gooderham M Poulin Y Mrowietz U Ferrandiz C . Efficacy and safety of apremilast, an oral phosphodiesterase 4 inhibitor, in patients with moderate-to-Severe plaque psoriasis over 52 weeks: a phase iii, randomized controlled trial (Esteem 2). Br J Dermatol (2015) 173(6):1387–99. doi: 10.1111/bjd.14164 26357944

[31] Reich K Gooderham M Green L Bewley A Zhang Z Khanskaya I . The efficacy and safety of apremilast, etanercept and placebo in patients with moderate-to-Severe plaque psoriasis: 52-week results from a phase iiib, randomized, placebo-controlled trial (Liberate). J Eur Acad Dermatol Venereol (2017) 31(3):507–17. doi: 10.1111/jdv.14015 PMC5363370 27768242

[32] Ohtsuki M Okubo Y Komine M Imafuku S Day RM Chen P . Apremilast, an oral phosphodiesterase 4 inhibitor, in the treatment of Japanese patients with moderate to severe plaque psoriasis: efficacy, safety and tolerability results from a phase 2b randomized controlled trial. J Dermatol (2017) 44(8):873–84. doi: 10.1111/1346-8138.13829 PMC5573969 28391657

[33] Strober B Bagel J Lebwohl M Stein Gold L Jackson JM Chen R . Efficacy and safety of apremilast in patients with moderate plaque psoriasis with lower bsa: week 16 results from the unveil study. J Drugs Dermatol (2017) 16(8):801–8. 28809995

[34] Armstrong AW Gooderham M Warren RB Papp KA Strober B Thaçi D . Deucravacitinib versus placebo and apremilast in moderate to severe plaque psoriasis: efficacy and safety results from the 52-week, randomized, double-blinded, placebo-controlled phase 3 poetyk pso-1 trial. J Am Acad Dermatol (2023) 88(1):29–39. doi: 10.1016/j.jaad.2022.07.002 35820547

[35] Papp K Gordon K Thaçi D Morita A Gooderham M Foley P . Phase 2 trial of selective tyrosine kinase 2 inhibition in psoriasis. N Engl J Med (2018) 379(14):1313–21. doi: 10.1056/NEJMoa1806382 30205746

[36] Strober B Thaçi D Sofen H Kircik L Gordon KB Foley P . Deucravacitinib versus placebo and apremilast in moderate to severe plaque psoriasis: efficacy and safety results from the 52-week, randomized, double-blinded, phase 3 program for evaluation of tyk2 inhibitor psoriasis second trial. J Am Acad Dermatol (2023) 88(1):40–51. doi: 10.1016/j.jaad.2022.08.061 36115523

[37] Amgen . Prescribing information for otezla (Apremilast) (2014). Available at: https://www.pi.amgen.com/-/media/Project/Amgen/Repository/pi-amgen-com/Otezla/otezla_pi_english.pdf (Accessed 2023 May 8).

[38] Zhang L Guo L Wang L and Jiang X . The efficacy and safety of tofacitinib, peficitinib, solcitinib, baricitinib, abrocitinib and deucravacitinib in plaque psoriasis - a network meta-analysis. J Eur Acad Dermatol Venereol (2022) 36(11):1937–46. doi: 10.1111/jdv.18263 35608188

[39] Wcisło-Dziadecka D Zbiciak-Nylec M Brzezińska-Wcisło L Bebenek K Kaźmierczak A . Newer treatments of psoriasis regarding il-23 inhibitors, phosphodiesterase 4 inhibitors, and janus kinase inhibitors. Dermatol Ther (2017) 30(6):e12555. doi: 10.1111/dth.12555 28994166

[40] Schafer P . Apremilast mechanism of action and application to psoriasis and psoriatic arthritis. Biochem Pharmacol (2012) 83(12):1583–90. doi: 10.1016/j.bcp.2012.01.001 22257911

[41] Pincelli C Schafer PH French LE Augustin M Krueger JG . Mechanisms underlying the clinical effects of apremilast for psoriasis. J Drugs Dermatol (2018) 17(8):835–40. 30124722

[42] Clark JD Flanagan ME Telliez JB . Discovery and development of janus kinase (Jak) inhibitors for inflammatory diseases. J Med Chem (2014) 57(12):5023–38. doi: 10.1021/jm401490p 24417533

[43] Banerjee S Biehl A Gadina M Hasni S Schwartz DM . Jak-stat signaling as a target for inflammatory and autoimmune diseases: current and future prospects. Drugs (2017) 77(5):521–46. doi: 10.1007/s40265-017-0701-9 PMC7102286 28255960

[44] Solimani F Meier K Ghoreschi K . Emerging topical and systemic jak inhibitors in dermatology. Front Immunol (2019) 10:2847. doi: 10.3389/fimmu.2019.02847 31849996 PMC6901833

[45] Lé AM Puig L Torres T . Deucravacitinib for the treatment of psoriatic disease. Am J Clin Dermatol (2022) 23(6):813–22. doi: 10.1007/s40257-022-00720-0 PMC9372960 35960487

[46] Jo CE Gooderham M Beecker J . Tyk 2 inhibitors for the treatment of dermatologic conditions: the evolution of jak inhibitors. Int J Dermatol (2022) 61(2):139–47. doi: 10.1111/ijd.15605 33929045

[47] Cline A Cardwell LA Feldman SR . Advances in treating psoriasis in the elderly with small molecule inhibitors. Expert Opin Pharmacother (2017) 18(18):1965–73. doi: 10.1080/14656566.2017.1409205 29171774

