# Supplementary figures and images for: Oral small-molecule tyrosine kinase 2 and phosphodiesterase 4 inhibitors in plaque psoriasis: a network meta-analysis
Source: Front Immunol. 2023 Jun 2;14:1180170. doi: 10.3389/fimmu.2023.1180170 (PMC10272578; doi:10.3389/fimmu.2023.1180170)

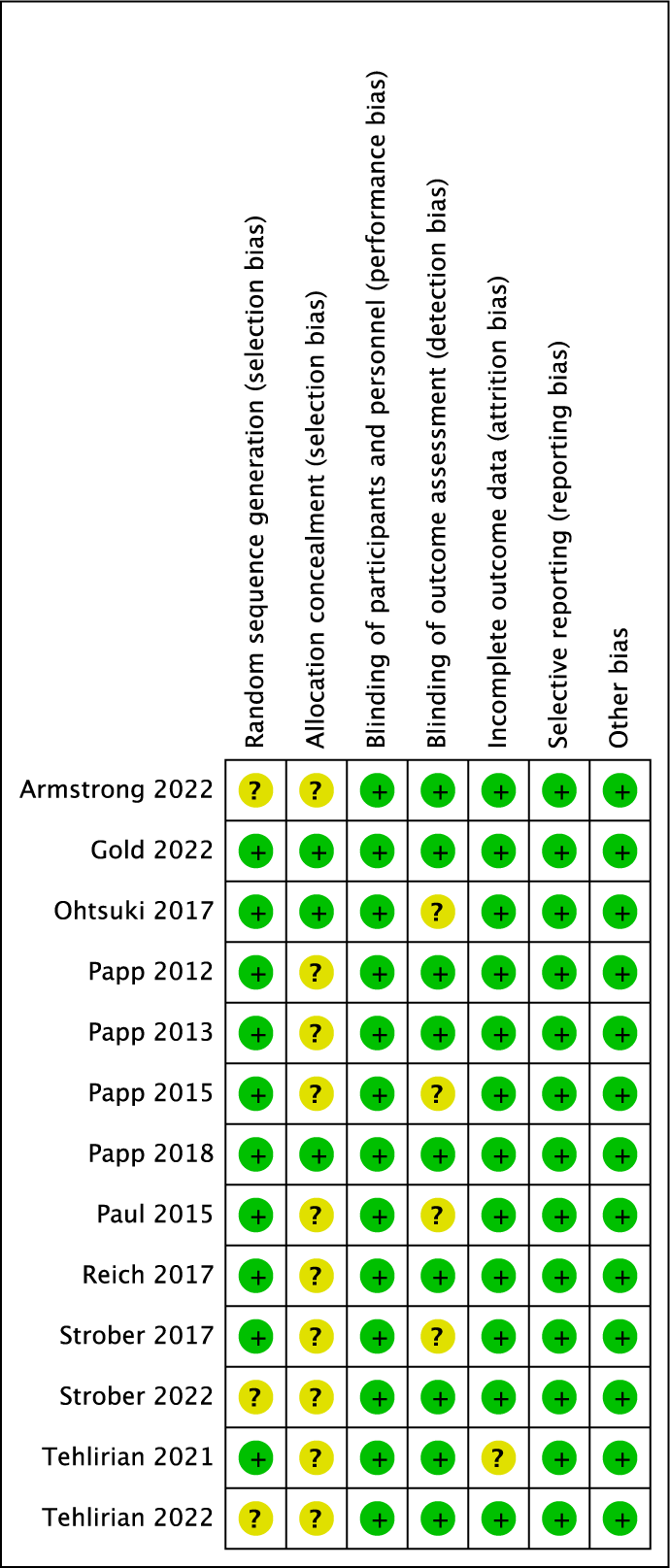

Supplement: Supplementary Figure 1 — Summary of risk of bias: review authors’ judgements about each risk of bias item for each included study. [file Image_1.tif]

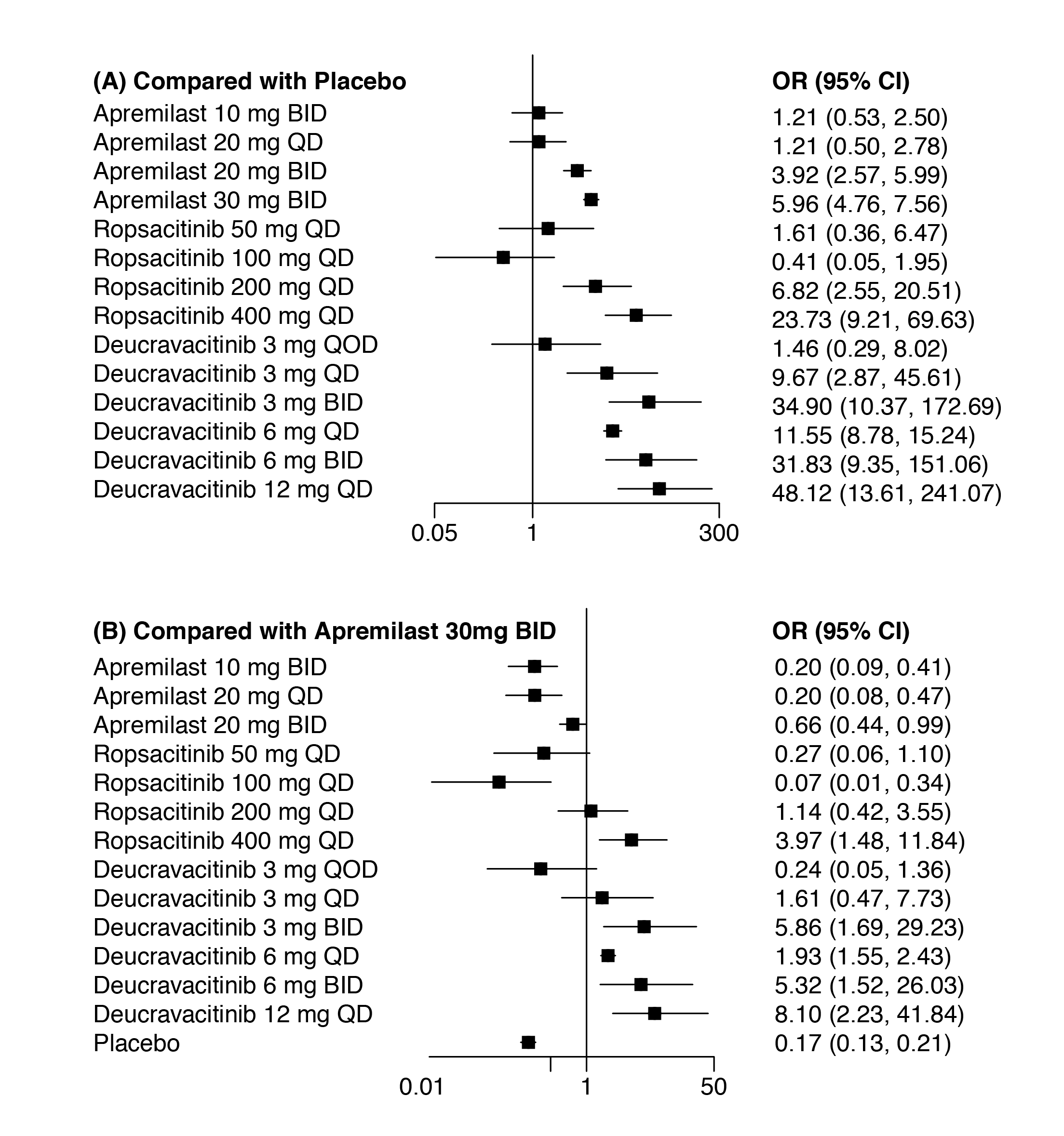

Supplement: Supplementary Figure 2 — Forest plot of the PASI-75 response. [file Image_2.tif]

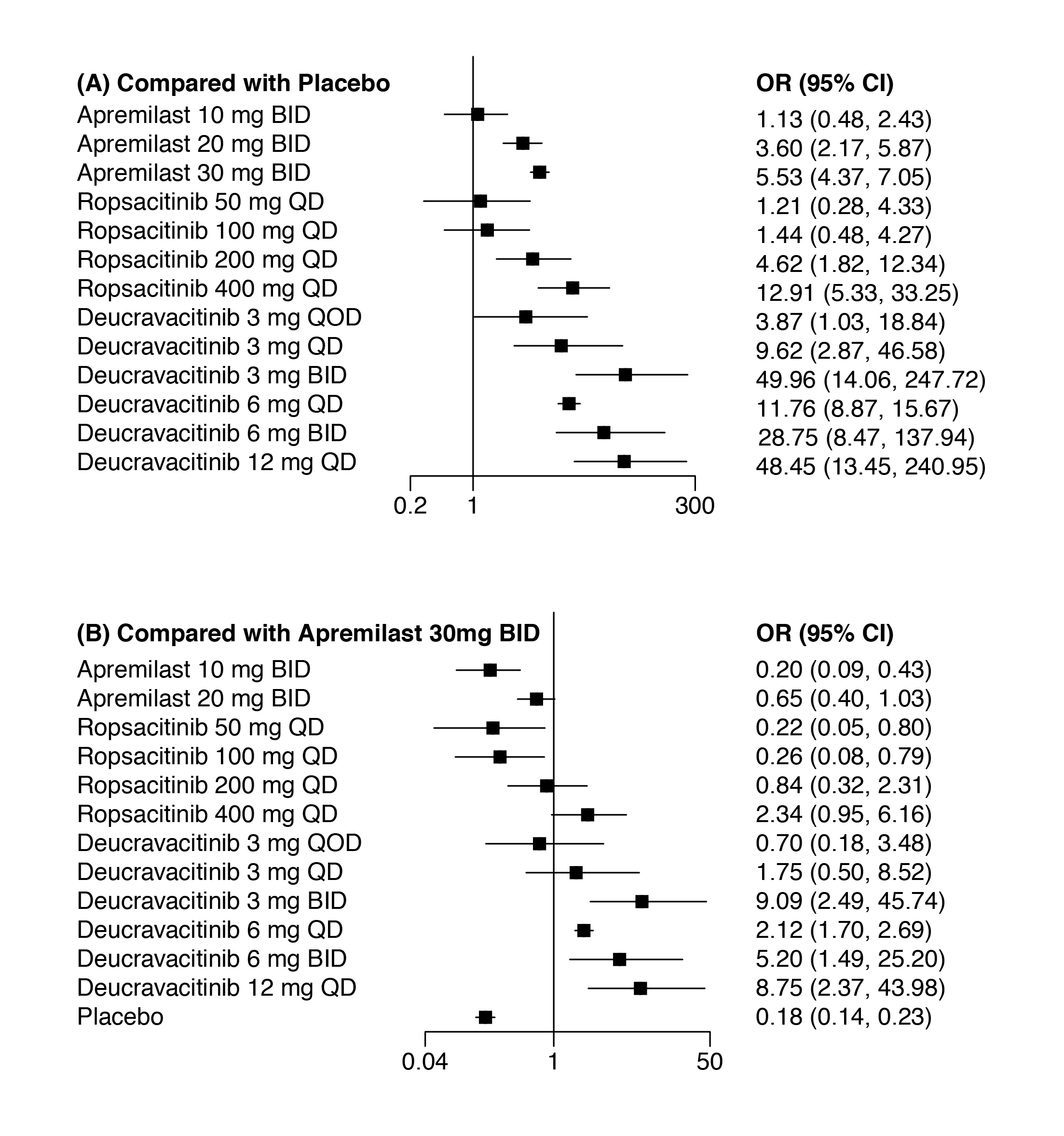

Supplement: Supplementary Figure 3 — Forest plot of the PGA 0/1 response. [file Image_3.tif]

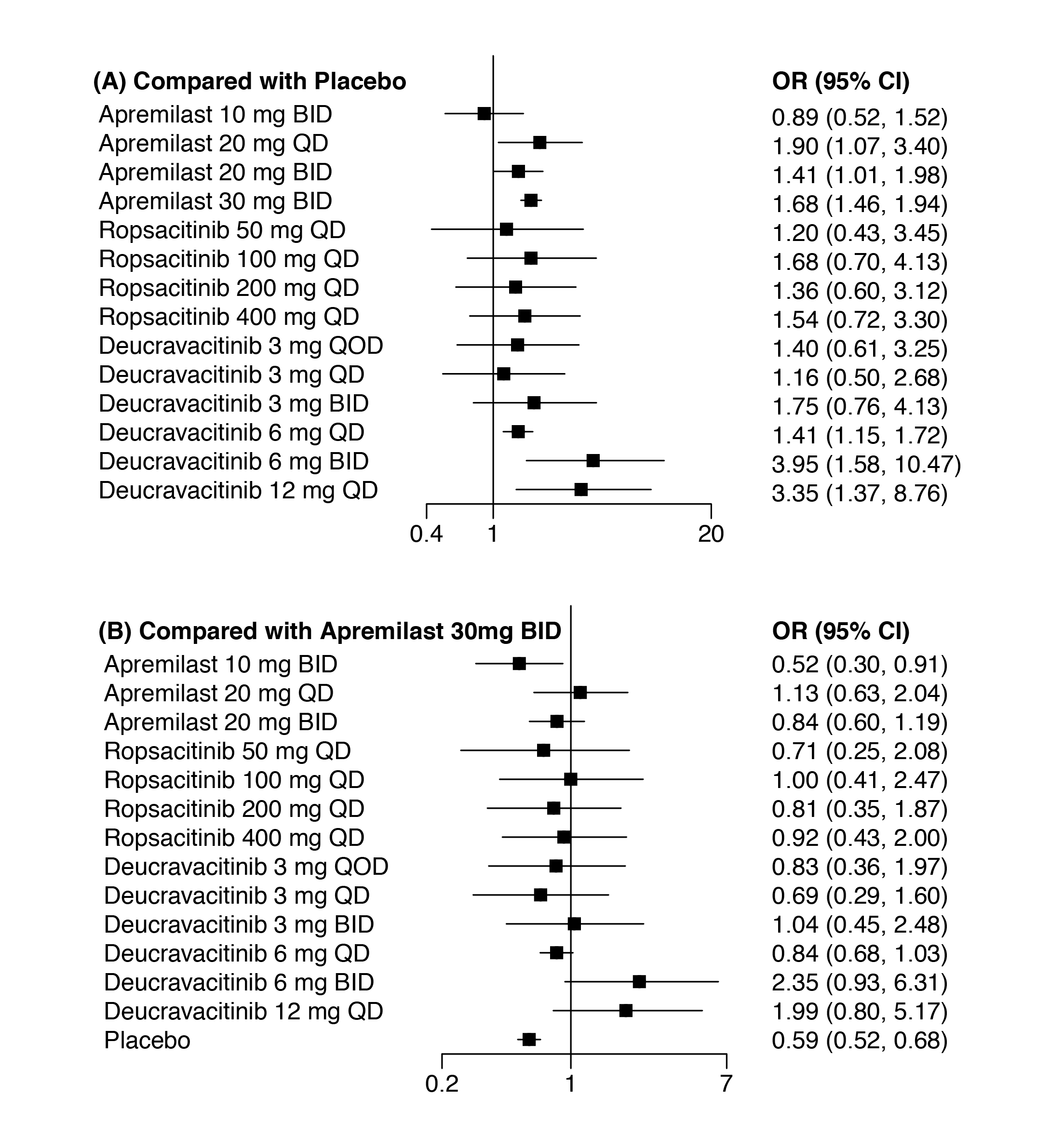

Supplement: Supplementary Figure 4 — Forest plot of the incidence of AEs. [file Image_4.tif]
