# Supplementary material for: Oral small-molecule tyrosine kinase 2 and phosphodiesterase 4 inhibitors in plaque psoriasis: a network meta-analysis
Source: Front Immunol. 2023 Jun 2;14:1180170. doi: 10.3389/fimmu.2023.1180170 (PMC10272578; doi:10.3389/fimmu.2023.1180170)
Supplement: Supplementary file 5 [file Table_1.docx]

**Table S1A** The search strategy in PubMed database

| **Topic** | **Number** | **Query** | **No. of studies** |
| --- | --- | --- | --- |
| Patient/disease | #1 | ("Psoriasis"[Mesh]) OR (((((Psoriases[Title/Abstract]) OR (Pustulosis Palmaris et Plantaris[Title/Abstract])) OR (Palmoplantaris Pustulosis[Title/Abstract]))) OR (Pustular Psoriasis of Palms and Soles[Title/Abstract])) | 47623 |
| PDE4 inhibitors | #2 | "Phosphodiesterase 4 Inhibitors"[MeSH Terms] OR "inhibitor phosphodiesterase 4"[Title/Abstract] OR "pde 4 inhibitor"[Title/Abstract] OR "inhibitor pde 4"[Title/Abstract] OR "pde 4 inhibitor"[Title/Abstract] OR "phosphodiesterase type 4 inhibitors"[Title/Abstract] OR "phosphodiesterase type 4 inhibitor"[Title/Abstract] OR "pde 4 inhibitors"[Title/Abstract] OR "inhibitors pde 4"[Title/Abstract] OR "pde 4 inhibitors"[Title/Abstract] OR "inhibitors pde4"[Title/Abstract] OR "phosphodiesterase 4 inhibitor"[Title/Abstract] OR "4 inhibitor phosphodiesterase"[Title/Abstract] OR "inhibitor phosphodiesterase 4"[Title/Abstract] OR "pde4 inhibitor"[Title/Abstract] OR "inhibitor pde4"[Title/Abstract] | 2407 |
| TYK2 inhibitors | #3 | "TYK2 Kinase"[MeSH Terms] OR "kinase tyk2"[Title/Abstract] OR "non receptor tyrosine protein kinase tyk2"[Title/Abstract] OR "non receptor tyrosine protein kinase tyk2"[Title/Abstract] OR "tyrosine kinase 2"[Title/Abstract] OR "TYK2"[Title/Abstract] | 2382 |
| Apremilast | #4 | "apremilast"[Supplementary Concept] OR "Otezla"[Title/Abstract] OR "CC-10004"[Title/Abstract] OR "CC10004"[Title/Abstract] OR "CC 10004"[Title/Abstract] | 619 |
| Deucravacitinib | #5 | "deucravacitinib"[Supplementary Concept] OR "BMS-986165"[Title/Abstract] | 35 |
| Brepocitinib | #6 | "Brepocitinib"[Title/Abstract] OR "PF-06700841"[Supplementary Concept] | 34 |
| Ropsacitinib | #7 | "ropsacitinib"[Supplementary Concept] OR "PF-06826647"[Title/Abstract] | 10 |
| Intervention | #8 | ((((("Phosphodiesterase 4 Inhibitors"[MeSH Terms] OR "inhibitor phosphodiesterase 4"[Title/Abstract] OR "pde 4 inhibitor"[Title/Abstract] OR "inhibitor pde 4"[Title/Abstract] OR "pde 4 inhibitor"[Title/Abstract] OR "phosphodiesterase type 4 inhibitors"[Title/Abstract] OR "phosphodiesterase type 4 inhibitor"[Title/Abstract] OR "pde 4 inhibitors"[Title/Abstract] OR "inhibitors pde 4"[Title/Abstract] OR "pde 4 inhibitors"[Title/Abstract] OR "inhibitors pde4"[Title/Abstract] OR "phosphodiesterase 4 inhibitor"[Title/Abstract] OR "4 inhibitor phosphodiesterase"[Title/Abstract] OR "inhibitor phosphodiesterase 4"[Title/Abstract] OR "pde4 inhibitor"[Title/Abstract] OR "inhibitor pde4"[Title/Abstract]) OR ("TYK2 Kinase"[MeSH Terms] OR "kinase tyk2"[Title/Abstract] OR "non receptor tyrosine protein kinase tyk2"[Title/Abstract] OR "non receptor tyrosine protein kinase tyk2"[Title/Abstract] OR "tyrosine kinase 2"[Title/Abstract] OR "TYK2"[Title/Abstract])) OR ("apremilast"[Supplementary Concept] OR "Otezla"[Title/Abstract] OR "CC-10004"[Title/Abstract] OR "CC10004"[Title/Abstract] OR "CC-10004"[Title/Abstract])) OR ("deucravacitinib"[Supplementary Concept] OR "BMS-986165"[Title/Abstract])) OR ("Brepocitinib"[Title/Abstract] OR "PF-06700841"[Supplementary Concept])) OR ("ropsacitinib"[Supplementary Concept] OR "PF-06826647"[Title/Abstract]) | 5191 |
| Study type | #9 | "randomized controlled trial"[Publication Type] OR "randomized"[Title/Abstract] OR "placebo"[Title/Abstract] | 1005675 |
| Final seach terms | #10 | ((("Psoriasis"[Mesh]) OR (((((Psoriases[Title/Abstract]) OR (Pustulosis Palmaris et Plantaris[Title/Abstract])) OR (Palmoplantaris Pustulosis[Title/Abstract]))) OR (Pustular Psoriasis of Palms and Soles[Title/Abstract]))) AND (((((("Phosphodiesterase 4 Inhibitors"[MeSH Terms] OR "inhibitor phosphodiesterase 4"[Title/Abstract] OR "pde 4 inhibitor"[Title/Abstract] OR "inhibitor pde 4"[Title/Abstract] OR "pde 4 inhibitor"[Title/Abstract] OR "phosphodiesterase type 4 inhibitors"[Title/Abstract] OR "phosphodiesterase type 4 inhibitor"[Title/Abstract] OR "pde 4 inhibitors"[Title/Abstract] OR "inhibitors pde 4"[Title/Abstract] OR "pde 4 inhibitors"[Title/Abstract] OR "inhibitors pde4"[Title/Abstract] OR "phosphodiesterase 4 inhibitor"[Title/Abstract] OR "4 inhibitor phosphodiesterase"[Title/Abstract] OR "inhibitor phosphodiesterase 4"[Title/Abstract] OR "pde4 inhibitor"[Title/Abstract] OR "inhibitor pde4"[Title/Abstract]) OR ("TYK2 Kinase"[MeSH Terms] OR "kinase tyk2"[Title/Abstract] OR "non receptor tyrosine protein kinase tyk2"[Title/Abstract] OR "non receptor tyrosine protein kinase tyk2"[Title/Abstract] OR "tyrosine kinase 2"[Title/Abstract] OR "TYK2"[Title/Abstract])) OR ("apremilast"[Supplementary Concept] OR "Otezla"[Title/Abstract] OR "CC-10004"[Title/Abstract] OR "CC10004"[Title/Abstract] OR "CC-10004"[Title/Abstract])) OR ("deucravacitinib"[Supplementary Concept] OR "BMS-986165"[Title/Abstract])) OR ("Brepocitinib"[Title/Abstract] OR "PF-06700841"[Supplementary Concept])) OR ("ropsacitinib"[Supplementary Concept] OR "PF-06826647"[Title/Abstract]))) AND ("randomized controlled trial"[Publication Type] OR "randomized"[Title/Abstract] OR "placebo"[Title/Abstract]) | 116 |

**Table S1B** The search strategy in Embase database

| **Topic** | **Number** | **Query** | **No. of studies** |
| --- | --- | --- | --- |
| Patient/disease | #1 | 'psoriasis'/exp OR 'psoriasis' | 121424 |
|  | #2 | psoriases:ab,ti | 30 |
|  | #3 | 'pustulosis palmaris et plantaris':ab,ti | 220 |
|  | #4 | 'palmoplantaris pustulosis':ab,ti | 5 |
|  | #5 | 'pustular psoriasis of palms and soles':ab,ti | 3 |
|  | #6 | #1 OR #2 OR #3 OR #4 OR #5 | 121468 |
| PDE4 inhibitors | #7 | 'phosphodiesterase iv inhibitor'/exp OR 'phosphodiesterase iv inhibitor' | 13827 |
|  | #8 | 'inhibitor, phosphodiesterase 4':ab,ti | 1 |
|  | #9 | 'pde-4 inhibitor':ab,ti | 231 |
|  | #10 | 'inhibitor, pde-4':ab,ti | 4 |
|  | #11 | 'pde 4 inhibitor':ab,ti | 231 |
|  | #12 | 'phosphodiesterase type 4 inhibitors':ab,ti | 32 |
|  | #13 | 'phosphodiesterase type 4 inhibitor':ab,ti | 111 |
|  | #14 | 'pde-4 inhibitors':ab,ti | 180 |
|  | #15 | 'inhibitors, pde-4':ab,ti | 6 |
|  | #16 | 'pde 4 inhibitors':ab,ti | 179 |
|  | #17 | 'pde4 inhibitors':ab,ti | 1099 |
|  | #18 | 'inhibitors, pde4':ab,ti | 28 |
|  | #19 | 'phosphodiesterase 4 inhibitor':ab,ti | 1044 |
|  | #20 | '4 inhibitor, phosphodiesterase':ab,ti | 2 |
|  | #21 | 'inhibitor, phosphodiesterase 4':ab,ti | 1 |
|  | #22 | 'pde4 inhibitor':ab,ti | 1462 |
|  | #23 | 'inhibitor, pde4':ab,ti | 10 |
|  | #24 | #7 OR #8 OR #9 OR #10 OR #11 OR #12 OR #13 OR #14 OR #15 OR #16 OR #17 OR #18 OR #19 OR #20 OR #21 OR #22 OR #23 | 14140 |
| TYK2 inhibitors | #25 | 'protein kinase tyk2'/exp OR 'protein kinase tyk2' | 1264 |
|  | #26 | 'kinase, tyk2':ab,ti | 73 |
|  | #27 | 'non-receptor tyrosine-protein kinase tyk2':ab,ti | 2 |
|  | #28 | 'non receptor tyrosine-protein kinase tyk2':ab,ti | 2 |
|  | #29 | 'tyrosine kinase 2':ab,ti | 1566 |
|  | #30 | 'tyk2':ab,ti | 1986 |
|  | #31 | #25 OR #26 OR #27 OR #28 OR #29 OR #30 | 3696 |
| Apremilast | #32 | 'apremilast'/exp OR 'apremilast' | 3645 |
|  | #33 | 'otezla':ab,ti | 37 |
|  | #34 | 'cc-10004':ab,ti | 13 |
|  | #35 | 'cc10004':ab,ti | 12 |
|  | #36 | 'cc 10004':ab,ti | 13 |
|  | #37 | #32 OR #33 OR #34 OR #35 OR #36 | 3646 |
| Deucravacitinib | #38 | 'deucravacitinib'/exp OR 'deucravacitinib' | 252 |
|  | #39 | 'bms-986165':ab,ti | 48 |
|  | #40 | #38 OR #39 | 280 |
| Brepocitinib | #41 | 'brepocitinib'/exp OR 'brepocitinib' | 130 |
|  | #42 | 'pf-06700841':ab,ti | 21 |
|  | #43 | #41 OR #42 | 137 |
| Ropsacitinib | #44 | 'ropsacitinib'/exp OR 'ropsacitinib' | 28 |
|  | #45 | 'pf-06826647':ab,ti | 11 |
|  | #46 | #44 OR #45 | 29 |
| Intervention | #47 | #24 OR #31 OR #37 OR #40 OR #43 OR #46 | 18000 |
| Study type | #48 | 'randomized controlled trial'/exp OR 'randomized controlled trial' | 1033120 |
| Final seach terms | #49 | #6 AND #47 AND #48 AND [embase]/lim | 683 |

**Table S1C** The search strategy in Cochrane Library (Trials)

| **Topic** | **Number** | **Query** | **No. of studies** |
| --- | --- | --- | --- |
| Patient/disease | #1 | MeSH descriptor: [Psoriasis] explode all trees | 4545 |
|  | #2 | (Psoriases):ti,ab,kw (Word variations have been searched) | 8 |
|  | #3 | (Pustulosis of Palms and Soles):ti,ab,kw (Word variations have been searched) | 45 |
|  | #4 | (Pustulosis Palmaris et Plantaris):ti,ab,kw (Word variations have been searched) | 5 |
|  | #5 | (Palmoplantaris Pustulosis):ti,ab,kw (Word variations have been searched) | 102 |
|  | #6 | (Pustular Psoriasis of Palms and Soles):ti,ab,kw (Word variations have been searched) | 30 |
|  | #7 | #1 OR #2 OR #3 OR #4 OR #5 OR #6 | 4635 |
| PDE4 inhibitors | #8 | MeSH descriptor: [Phosphodiesterase 4 Inhibitors] explode all trees | 133 |
|  | #9 | (Inhibitor, Phosphodiesterase 4):ti,ab,kw (Word variations have been searched) | 1720 |
|  | #10 | (PDE-4 Inhibitor):ti,ab,kw (Word variations have been searched) | 47 |
|  | #11 | (Inhibitor, PDE-4):ti,ab,kw (Word variations have been searched) | 47 |
|  | #12 | (PDE 4 Inhibitor):ti,ab,kw (Word variations have been searched) | 249 |
|  | #13 | (Phosphodiesterase Type 4 Inhibitors):ti,ab,kw (Word variations have been searched) | 520 |
|  | #14 | (PDE-4 Inhibitors):ti,ab,kw (Word variations have been searched) | 47 |
|  | #15 | (Inhibitors, PDE-4):ti,ab,kw (Word variations have been searched) | 47 |
|  | #16 | (PDE 4 Inhibitors):ti,ab,kw (Word variations have been searched) | 249 |
|  | #17 | (PDE4 Inhibitors):ti,ab,kw (Word variations have been searched) | 205 |
|  | #18 | (Inhibitors, PDE4):ti,ab,kw (Word variations have been searched) | 205 |
|  | #19 | (Phosphodiesterase 4 Inhibitor):ti,ab,kw (Word variations have been searched) | 1720 |
|  | #20 | (4 Inhibitor, Phosphodiesterase):ti,ab,kw | 1165 |
|  | #21 | (Inhibitor, Phosphodiesterase 4):ti,ab,kw | 1165 |
|  | #22 | (PDE4 Inhibitor):ti,ab,kw | 193 |
|  | #23 | (Inhibitor, PDE4):ti,ab,kw | 193 |
|  | #24 | #8 OR #9 OR #10 OR #11 OR #12 OR #13 OR #14 OR #15 OR #16 OR #17 OR #18 OR #19 OR #20 OR #21 OR #22 OR #23 | 1858 |
| TYK2 inhibitors | #25 | MeSH descriptor: [TYK2 Kinase] explode all trees | 14 |
|  | #26 | (Kinase, TYK2):ti,ab,kw | 107 |
|  | #27 | (Non-Receptor Tyrosine-Protein Kinase TYK2):ti,ab,kw | 0 |
|  | #28 | (Non Receptor Tyrosine Protein Kinase TYK2):ti,ab,kw | 0 |
|  | #29 | (Tyrosine Kinase 2):ti,ab,kw | 3246 |
|  | #30 | (TYK2):ti,ab,kw | 133 |
|  | #31 | #25 OR #26 OR #27 OR #28 OR #29 OR #30 | 3286 |
| Apremilast | #32 | (apremilat):ti,ab,kw | 0 |
|  | #33 | (Otezla):ti,ab,kw | 19 |
|  | #34 | (CC-10004):ti,ab,kw | 42 |
|  | #35 | (CC10004):ti,ab,kw | 0 |
|  | #36 | (CC 10004):ti,ab,kw | 42 |
|  | #37 | #32 OR #33 OR #34 OR #35 OR #36 | 56 |
| Deucravacitinib | #38 | (deucravacitinib):ti,ab,kw | 72 |
|  | #39 | (BMS-986165):ti,ab,kw | 74 |
|  | #40 | #38 OR #39 | 136 |
| Brepocitinib | #41 | (Brepocitinib):ti,ab,kw | 16 |
|  | #42 | (PF-06700841):ti,ab,kw | 31 |
|  | #43 | #41 OR #42 | 46 |
| Ropsacitinib | #44 | (ropsacitinib):ti,ab,kw | 0 |
|  | #45 | (PF-06826647):ti,ab,kw | 8 |
|  | #46 | #44 OR #45 | 8 |
| Intervention | #47 | #24 OR #31 OR #37 OR #40 OR #43 OR #46 | 5269 |
| Study type | #48 | (randomized controlled trial):pt | 0 |
|  | #49 | (randomized):ti,ab,kw | 1080166 |
|  | #50 | (placebo):ti,ab,kw | 355582 |
|  | #51 | #48 OR #49 OR #50 | 1197181 |
| Final seach terms | #52 | #7 AND #47 AND #51 | 91 |
