# Supplementary material for: Oral small-molecule tyrosine kinase 2 and phosphodiesterase 4 inhibitors in plaque psoriasis: a network meta-analysis
Source: Front Immunol. 2023 Jun 2;14:1180170. doi: 10.3389/fimmu.2023.1180170 (PMC10272578; doi:10.3389/fimmu.2023.1180170)
Supplement: Supplementary file 6 [file Table_2.docx]

**Table S2A.** Fixed model vs Random model for all trails (PASI) compared with placebo

| Treatment | Fixed-effect model | Random-effect model |
| --- | --- | --- |
|  | OR(95%Crl) | OR(95%Crl) |
| Apremilast 10mg BID | 1.21 (0.53, 2.5) | 1.24 (0.5, 2.87) |
| Apremilast 20mg QD | 1.21 (0.5, 2.78) | 1.2 (0.43, 3.1) |
| Apremilast 20mg BID | 3.92 (2.57, 5.99) | 3.91 (2.4, 6.48) |
| Apremilast 30mg BID | 5.96 (4.76, 7.56) | 5.91 (4.39, 7.98) |
| Ropsacitinib 50mg QD | 1.61 (0.36, 6.47) | 1.58 (0.33, 6.87) |
| Ropsacitinib 100mg QD | 0.41 (0.05, 1.95) | 0.42 (0.06, 2.26) |
| Ropsacitinib 200mg QD | 6.82 (2.55, 20.51) | 6.76 (2.3, 22.43) |
| Ropsacitinib 400mg QD | 23.73 (9.21, 69.63) | 24.35 (8.62, 79.84) |
| Deucravacitinib 3mg QOD | 1.46 (0.29, 8.02) | 1.38 (0.29, 8.5) |
| Deucravacitinib 3mg QD | 9.67 (2.87, 45.61) | 9.54 (2.66, 49.36) |
| Deucravacitinib 3mg BID | 34.9 (10.37, 172.69) | 35.11 (9.75, 182.17) |
| Deucravacitinib 6mg QD | 11.55 (8.78, 15.24) | 11.53 (7.46, 17.82) |
| Deucravacitinib 6mg BID | 31.83 (9.35, 151.06) | 31.4 (8.44, 165) |
| Deucravacitinib 12mg QD | 48.12 (13.61, 241.07) | 47.94 (13.11, 258.68) |

**Table S2B.** Fixed model vs Random model for all trails (PGA) compared with placebo

| Treatment | Fixed-effect model | Random-effect model |
| --- | --- | --- |
|  | OR(95%Crl) | OR(95%Crl) |
| Apremilast 10mg BID | 1.13 (0.48, 2.43) | 1.11 (0.43, 2.51) |
| Apremilast 20mg BID | 3.6 (2.17, 5.87) | 3.58 (2.06, 6.18) |
| Apremilast 30mg BID | 5.53 (4.37, 7.05) | 5.52 (4.23, 7.24) |
| Ropsacitinib 50mg QD | 1.21 (0.28, 4.33) | 1.13 (0.25, 4.26) |
| Ropsacitinib 100mg QD | 1.44 (0.48, 4.27) | 1.49 (0.49, 4.44) |
| Ropsacitinib 200mg QD | 4.62 (1.82, 12.34) | 4.66 (1.76, 13.27) |
| Ropsacitinib 400mg QD | 12.91 (5.33, 33.25) | 12.96 (4.99, 34.97) |
| Deucravacitinib 3mg QOD | 3.87 (1.03, 18.84) | 3.79 (0.98, 19.23) |
| Deucravacitinib 3mg QD | 9.62 (2.87, 46.58) | 9.76 (2.76, 46.67) |
| Deucravacitinib 3mg BID | 49.96 (14.06, 247.72) | 49.38 (13.5, 254.55) |
| Deucravacitinib 6mg QD | 11.76 (8.87, 15.67) | 11.88 (8.32, 17.29) |
| Deucravacitinib 6mg BID | 28.75 (8.47, 137.94) | 28.23 (8.2, 138.28) |
| Deucravacitinib 12mg QD | 48.45 (13.45, 240.95) | 47.47 (13.23, 238.65) |

**Table S2C.** Fixed model vs Random model for all trails (AE) compared with placebo

| Treatment | Fixed-effect model | Random-effect model |
| --- | --- | --- |
|  | OR(95%Crl) | OR(95%Crl) |
| Apremilast 10mg BID | 0.89 (0.52, 1.52) | 0.89 (0.47, 1.65) |
| Apremilast 20mg QD | 1.9 (1.07, 3.4) | 1.9 (0.98, 3.7) |
| Apremilast 20mg BID | 1.41 (1.01, 1.98) | 1.42 (0.96, 2.11) |
| Apremilast 30mg BID | 1.68 (1.46, 1.94) | 1.68 (1.38, 2.05) |
| Ropsacitinib 50mg QD | 1.2 (0.43, 3.45) | 1.24 (0.39, 3.79) |
| Ropsacitinib 100mg QD | 1.68 (0.7, 4.13) | 1.65 (0.65, 4.41) |
| Ropsacitinib 200mg QD | 1.36 (0.6, 3.12) | 1.34 (0.54, 3.24) |
| Ropsacitinib 400mg QD | 1.54 (0.72, 3.3) | 1.51 (0.66, 3.46) |
| Deucravacitinib 3mg QOD | 1.4 (0.61, 3.25) | 1.39 (0.56, 3.49) |
| Deucravacitinib 3mg QD | 1.16 (0.5, 2.68) | 1.17 (0.45, 2.88) |
| Deucravacitinib 3mg BID | 1.75 (0.76, 4.13) | 1.77 (0.68, 4.57) |
| Deucravacitinib 6mg QD | 1.41 (1.15, 1.72) | 1.41 (1.03, 1.97) |
| Deucravacitinib 6mg BID | 3.95 (1.58, 10.47) | 4.03 (1.48, 11.68) |
| Deucravacitinib 12mg QD | 3.35 (1.37, 8.76) | 3.38 (1.24, 9.61) |
